# Recurrent Dermatofibrosarcoma Protuberance: A Case Report

**DOI:** 10.31729/jnma.7187

**Published:** 2021-11-30

**Authors:** Shiv Raj Shah, Sujan Regmee, Dhiresh Kumar Maharjan, Prabin Bikram Thapa

**Affiliations:** 1 Department of Surgery, Kathmandu Medical College Teaching Hospital, Sinamangal, Kathmandu, Nepal

**Keywords:** *adjuvant radiotherapy, dermatofibrosarcoma*, *recurrence*, *skin grafting*

## Abstract

Dermatofibrosarcoma protuberance represents less than 0.1% of all tumors, treatment of which requires wide local excision (≥5cm) but recurrence is not rare. Here we present a 32-year male presented with a swelling of 15 × 6cm over the left lumbar region for which he underwent excision three years ago, the histopathological examination of the swelling, showed a malignant mesenchymal tumor and Immunohistochemistry features were suggestive of Dermatofibrosarcoma protuberance. After three years of interval, he again presented with complaints of swelling in the previously operated site for nine months and underwent excision of the mass with Split Thickness Skin Graft. Although the tumor was confined to the skin and subcutaneous tissue in the present case, the patient didn't undergo any adjuvant radiotherapy to avoid a possible relapse that would infiltrate deeper structures for the first time. Being a recurrent tumor, long-term follow-up is strongly recommended.

## INTRODUCTION

Dermatofibrosarcoma protuberance (DFSP) was first described by Darier and Ferrand in 1924, but the definition of "DFSP'' was established by Hoffman in 1925.^1^ It is a soft tissue sarcoma which is of low to intermediate grade and rare, being derived from the dermal layer of the skin. Originally present as a skin-colored plaque with possible dark red or blue discoloration which is painless. DFSP is a rare soft tissue neoplasm with low grade malignancy.^2^

The annual incidence is reported to be 0.8-4.5 cases per million in the USA. The most common location being the trunk (42-72%) followed by proximal extremities (20-30%), and head and neck (10-16%).^2^ We present a 32-year male presented with a swelling over the left lumbar region for which he underwent excision.

## CASE REPORT

**Figure 1:**
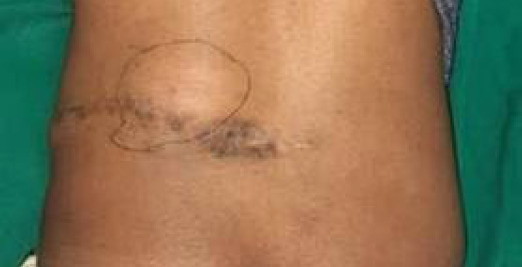
Pre-operative appearance of the mass.

A 32-year-old man presented with a mass located at the left loin, increasing in size associated with pricking pain for nine months (Figure 1). The patient also reported an increase in the size of the mass during the last nine months. The patient denied any recent weight loss, fever, night sweats, or chills. He gave a past history of similar swelling for which he underwent excisional biopsy three years ago. The histopathological examination of the excised mass three years ago showed a malignant mesenchymal tumor with a possible differential diagnosis of malignant peripheral nerve sheath tumor and leiomyosarcoma. The finding was confirmed by immunohistochemistry which showed features suggestive of dermatofibrosarcoma protuberance. Due to poor patient compliance, the patient did not take any adjuvant therapy postresection.

At this presentation on physical examination, two firms, painless, masses of 8 × 3cm and 6 × 3cm was noted on the left loin with approximately 20cm horizontal scar mark on the overlying skin with no sign of inflammation. There were no palpable cervical or axillary lymph nodes. There was neither personal nor familial history of any skin or soft tissue malignancy.

**Figure 2:**
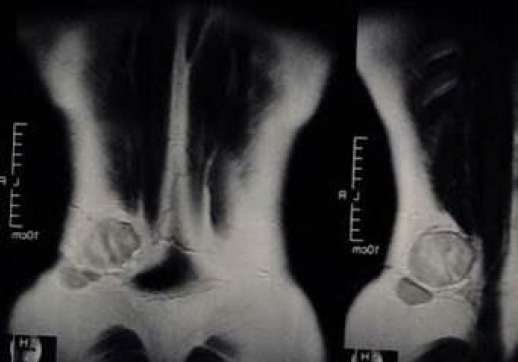
Pre-operative MRI picture.

On soft tissue ultrasound, a well-defined hypoechoic lesion of 55.4 × 40.1 × 39.2mm was noted within the subcutaneous plane of the left lumbar region, suspected to be a recurrence. Magnetic resonance imaging (MRI) of the left lower abdominal wall demonstrated two oval lesions of 8 × 4.7 × 5cm and 3.5 × 3 × 1.5cm in the subcutaneous fat plane at the left loin region (Figure 2).

**Figure 3:**
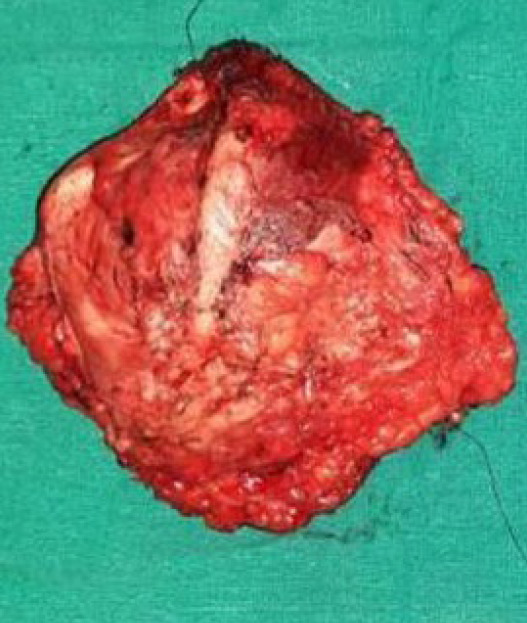
Excised mass of size 8 × 5cm from the left lumbar region.

An fine needle aspiration cytology of the tumor was done before planning for surgery which revealed the finding consistent with a tumor of the mesenchymal origin. All these findings were consistent with the recurrence of the previous lesion and the patient was prepared for surgery. Pre-anesthetic consultations were done and proper consent was taken from the patient. The patient underwent local excision of the tumor with a 3cm peripheral margin and the tissue defect that was created post-operatively was closed with a split-thickness skin graft taken from the left gluteal region. Mass of 8 × 5cm was excised and sent for HPE (Figure 3). Special effort was taken to avoid dissection beyond the midline over to the right side to prevent injury to the perforators. Wound dressing was inspected daily to look for any wound soakage and the graft was opened on the fifth postoperative day, the graft take was satisfactory on inspection. Subsequently, the drains were removed and the patient is doing well.

## DISCUSSION

The most frequently seen clinical scenario in dermatofibrosarcoma is its typical protuberant appearance. It can masquerade amorphea-like lesions with an appearance of an atrophic plaque at times.^3-6^ Lipoma, myofibroblastoma, fibromatosis, keloids, and hypertrophic scars are some of its differential diagnoses. The tumor is primarily located at the dermis with some variants that permeates to the subcutaneous plane.^7^ Less than 5% of the tumors present with metastatic disease, the most common site for metastasis is the lungs and the lymph nodes.^8^

Histopathological examination often supplemented with immunohistochemistry is the standard for diagnosing the condition. On ultrasound of the lesion hypoechoic or mixed hyperechoic appearance is seen with regular or irregular margin and a pseudopodialike projection such feature is also seen in conditions like lipoma, so diagnosis cannot be made based on the ultrasound findings.^9,10^ MRI is used widely to identify the extent of the disease and then plan for surgical resection but MRI also fails to distinguish the disease from other soft tissue conditions.

Wide local excision is the standard of care in these conditions. A margin of 3 to 5cm is the usual recommendation.^11,12^ Incidence of less than 5% of recurrence was noted in series where a 5cm margin was taken.^11^

The skin, subcutaneous tissue, and the underlying fascia are respected en-mass while exercising such a lesion. In conditions where the bone has involved the periosteum or even a part of the underlying bone needs to be excised to acquire a negative margin. On excision of a large tumor with adequate margin, the closure of the defect is often a challenge. The functional deformity is an issue when a large defect needs to be made while excising such tumors with adequate margin, cosmetic distortion is also a problem that needs to be addressed at times, depending on the location of the tumor. The Mohs micrographic surgery is advocated these days as an initial modality of treatment provided the disease is limited to a small area.

Radiotherapy for local control is used as an adjuvant treatment, in cases when the tumor is adequately excised and the margins are clear. However, it can also be used as the sole modality of care when the tumor is inoperable. Tumors with a positive margin post-resection are also treated with radiotherapy. A cure rate of up to 85% has been noted with the use of radiotherapy.^13^ The failure to take radiation after surgery due to poor patient compliance could be a possible cause of recurrence in our case.

Reports have shown the development of new lesions appearing even 5 years after treatment, which demands the need for twice-yearly clinical evaluation and monitoring of patients for an extended postoperative period.^14^

The origin of DFSP being dermis and tending to infiltrate underlying structures is the most common cutaneous sarcoma. It may infiltrate deeper structures like muscles, tendons, fascia and bone. In the present case, the tumor was confined to the skin and subcutaneous tissue, and our patient didn't undergo any adjuvant radiotherapy to avoid a possible relapse that would infiltrate deeper structures for the first time. Being a recurrent tumor, long-term follow-up is strongly recommended.
